# Experimental Research on Fatigue Behavior of Reinforced UHPC-NC Composite Beams under Cyclic Loading

**DOI:** 10.3390/ma17030747

**Published:** 2024-02-04

**Authors:** Jue Wang, Wenyu Ji, Wangwang Li, Tibo Zhao

**Affiliations:** 1 School of Civil Engineering and Architecture, Beijing Jiaotong University, Beijing 100044, China; 2 CCCC Highway Bridges National Engineering Research Centre Co., Ltd., Beijing 100088, China; 3 Railway Engineering Research Institute, China Academy of Railway Sciences Co., Ltd., Beijing 100081, China

**Keywords:** ultra-high-performance concrete (UHPC), composite beam, monotonic loading, cyclic loading, stiffness reduction coefficient

## Abstract

Ultra-high-performance concrete (UHPC), a new cement-based material that offers high mechanical strength and good durability, has been widely applied in construction and rehabilitation projects in recent years. An optimum bending system is achieved by positioning the UHPC layer at the bottom tensile zone of the composite beam and placing the normal-strength concrete (NC) layer at the upper compression zone, which is described as the UHPC-NC composite beam. The fatigue behavior of reinforced UHPC-NC composite beams was described in this study, with an emphasis on the effects of UHPC layer thickness and fatigue load level on the fatigue life of the beam, deformation of the interface between UHPC and NC layers, as well as the bending stiffness of the beam. A total of 9 reinforced UHPC-NC composite beams were tested under cyclic loading. The test variables include UHPC layer thicknesses (zero, 200, and 360 mm), reinforcement ratios (1.184% and 1.786%), and the upper load levels (0.39~0.65). The results showed that good bonding had been achieved without delamination between UHPC and NC layers prior to the final fatigue failure of the beam, and the bending stiffness of the composite beam experienced a three-stage reduction under cyclic loading. Furthermore, an equation was proposed to predict the stiffness reduction coefficient of UHPC-NC composite beams under cyclic loading.

## 1. Introduction

Even though conventional normal-strength concrete (NC) is commonly used in civil engineering due to its comprehensive performance and low cost, previous research has demonstrated that existing conventional reinforced concrete (RC) structures are structurally deficient under both repeated loading and environmental action [1,2,3,4]. Fatigue damage to RC structures manifests itself through the propagation of cracks and increased deformation over time and has become one of the most crucial problems for conventional RC structures. Especially in the case of structures mainly subjected to repeated loading, in such circumstances the ratio of external loads to dead loads (e.g., the traffic load to self-weight) is relatively high, and fatigue resistance has a great influence on the service life of them, such as the RC members, including bridges, airport pavements, concrete sleepers, machine foundations, etc. [5,6,7,8].

In view of the fact that the failure load under a fatigue load is far below that under a static load, the fatigue resistance of construction materials becomes of importance. In order to improve the performance of structures compared to conventional RC structures, various kinds of high-performance materials for members were studied [9,10]. As an advanced class of concrete material, Ultrahigh-performance concrete (UHPC) is a mixture of reactive powder concrete and fiber-reinforced materials, characterized by particle packing, a low water-to-binder ratio, and dense microstructure [11,12,13,14]. Because of its superior mechanical properties, extreme durability, and good energy dissipation characteristics, UHPC has attracted increasing applications in new construction and retrofitting projects [15,16,17,18], like high-rise buildings, bridges, railway structures, offshore structures, and so on. As a result, the UHPC consumed in civil engineering has gradually increased over the years; however, the majority of research has focused on repairing the existing RC beam. Now it is essential to optimize the application methods of UHPC in new structures. The UHPC-NC composite beam was developed to reduce the high initial cost of materials, which influences the application of UHPC in structures. In addition, a higher load-carrying capacity and increased cracking resistance are expected for the UHPC-NC composite beam compared with RC beams [19,20,21,22].

With respect to the fatigue damage of RC structures manifested in the inadequacy of loading carrying capacity, stiffness, and durability, applying a layer of UHPC to enhance the existing RC structures has shown a lot of promise to improve the carrying capacity and stiffness of the existing members, and the strengthened members further work as UHPC-NC composite members. For these composite members, the bonding property of the interface between the two parts of different materials plays a significant role in transferring load and improving their overall performance [23]. In the condition of inadequate interfacial bonding, the composite beam is prone to failure of interface debonding, which results in the underutilization of the strengthening layer. Compared with other strengthening plates that could be bonded with RC parts, such as FRP, CFRP, steel plate, etc. [24,25,26], there is a better bonding property between NC and UHPC parts due to both of them being cement-based composite materials [27,28,29,30], which could provide good deformation compatibility and interfacial durability and consequently retain or extend the service life of the composite beam.

Many studies on the behavior of UHPC-NC composite beams have been reported in recent years. Habel et al. [31] reported the increase in ultimate load-carrying capacity, stiffness, and cracking resistance of the ultra-high-performance fiber-reinforced concrete (UHPFRC)-RC composite members. Paschalis et al. [32] explored the influence of the reinforcement embedded in the UHPFRC layer on the flexural behavior of UHPFRC-NC composite beams, and a good interface characteristic between UHPFRC and NC parts was validated through push-off tests. Yin et al. [33] experimentally studied the impact of the longitudinal reinforcement embedded in the UHPC layer on the flexural and shear performance of the UHPC-NC composite beam. Test results showed that the overall stiffness and cracking resistance of the composite beam were improved by increasing the UHPC layer thicknesses. Al-Osta et al. [34] explored the flexural performance of UHPFRC-NC composite beams with three different configurations and two different interface treatments. They found that the stiffness of the composite beam was enhanced by the UHPFRC layer, and a good interface characteristic could be provided without surface preparation of the concrete substrate.

Comprehensive studies of the fatigue performance of NC beams were developed by researchers [35,36], and that of UHPC beams was studied as well [37,38]. For actual structures, UHPC members in service are subjected to the greater stress ranges attributed to its superior mechanical properties; as a consequence, UHPC members tend to be more sensitive to fatigue load levels in service life compared with NC members [39]. The effects of load range on the flexural performance of UHPC-NC composite beams under repeated loading were reported in published studies. Makita et al. [40] added a thin layer of UHPC to RC slabs and loaded them cyclically. The adopted maximum loads varied between 40% and 60% of the ultimate capacity of the beam; an obvious reduction in macrocracks for the composite beams was observed during bending fatigue tests. Al-Azzawi [41] conducted fatigue tests on RC beams strengthened with the unreinforced UHPC layer in three different load ranges, i.e., 3.5–46.9%, 3.5–61.0%, and 3.5–82.1% of the static load-carrying capacity. Although only one of the beams was loaded to failure, the results indicated that fatigue resistance was increased and the fatigue life was prolonged for the strengthened beams relative to the corresponding un-strengthened control beams. Murthy et al. [42] conducted experimental and numerical studies on RC beams rehabilitated by UHPFRC strips and found that a 10 mm-thick UHPFRC strip could effectively prolong the fatigue life and improve the bending stiffness of the retrofitted beam compared to the RC beam under fatigue loading. Li et al. [43] experimentally studied the fatigue performance of four different configurations of beams, including NC, NC-RUHPC, RC-RUHPC, and RUHPC composite beams, subjected to the same upper and lower loads. Test results showed that the improvements in bending stiffness and fatigue life of the beam were obvious in the RC-RUHPC composite beam. Ganesh et al. [44] reported the effects of three different thick (5, 10, and 15 mm) UHPC strips on the fatigue behavior of UHPC-RC composite beams with identical reinforcement ratios. The maximum deflection of the beam was found to decrease as the UHPC strip thickness increased. Very few flexural cracks were observed under cyclic loading and propagated through the interface between the UHPC strip and the RC beam, which finally expanded as the main crack.

Although the studies reported in the literature have demonstrated the contribution of the UHPC layer to the performance improvement of RC beams under bending, very limited research has been carried out on UHPC-NC composite beams under cyclic bending, especially research on the fatigue behavior of UHPC-NC composite beams with different UHPC layer thicknesses and reinforcement ratios, which is not well documented in the literature. The main objective of the present study is to investigate the effects of UHPC layer thickness, reinforcement ratio, and load level on the interface performance of the UHPC-NC composite beam under cyclic loading. This study could provide some additional useful information to describe the fatigue behavior of the UHPC-NC composite beam. The effects of the UHPC layer thickness, reinforcement ratio, and load level on the fatigue behavior of the composite beam were investigated in detail.

## 2. Bending Tests

### 2.1. Design of the Test Beam

**Figure 1:**
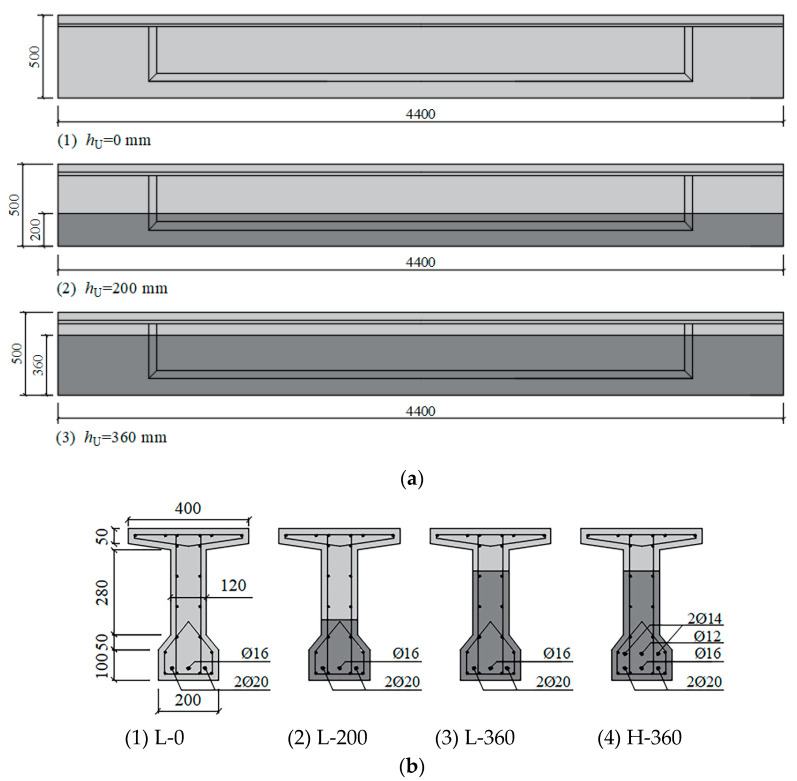
Dimension and configuration of beams (mm). (**a**) Front view; (**b**) Cross-sections.

Eleven 4400 mm long reinforced UHPC-NC composite T-beams were prepared for this investigation; all beams had the same overall dimensions. The dimensions and configuration of the beams are shown in Figure 1. Table 1 summarizes the parameters of beams tested under static and fatigue tests. Beams in series S were used as control beams and loaded monotonically to failure under four-point bending tests in order to evaluate the static behavior of beams. Beams in series L or series H were all subjected to cyclic loading.

The objective of the fatigue tests was to investigate the effects of different reinforcement ratios, thicknesses of the UHPC layer, and fatigue load levels on bending behavior under fatigue loading. The beams in fatigue tests were grouped into series with two different reinforcement ratios: the relatively low reinforcement ratio of 1.184% was denoted as series L, and the higher reinforcement ratio of 1.786% was denoted as series H. Three different thicknesses of UHPC layer (*h*_U_ = 0, 200, and 360 mm) were provided in series L; a 360 mm thick UHPC layer was used in series H. In addition, various fatigue loads were applied to the beams in these two series. Consequently, the beams were labeled by the parameters previously mentioned according to the notation, H/L-X-Y: H/L represented the higher or lower reinforcement ratio; X represented the UHPC layer thickness; Y represented the upper load level (the ratio of the upper load limit to the ultimate carrying capacity) for beams under fatigue loading, whereas S was indicated under static loading.

### 2.2. Materials

The detailed composition and mass ratios of NC and UHPC are given in Table 2. For the concrete, Type-I ordinary Portland cement (42.5), mineral powder, natural river sands, and crushed stone with a maximum particle size of 15 mm were used in the present investigation. Most UHPCs are designed without stone; however, the appropriate type and content of coarse aggregate can be used to improve the elastic modulus, reduce autogenous shrinkage for UHPC, and further develop a more economic and workable UHPC [45]. For this reason, the UHPC in this study consisted of Type-I ordinary Portland cement (42.5), fine silica, quartz sands, and crushed stone ranging in size from 5 to 10 mm, as well as straight steel fibers. The properties of steel fiber are provided in Table 3.

Six batches of NC and UHPC were used in the project for these eleven beams due to the limited capacity of the concrete mixer. The mechanical properties of the NC and UHPC used can be seen in Table 4. The average compressive strength of NC and UHPC was tested using 150 mm and 100 mm cubes, respectively, whereas the average flexural tensile strength was obtained using 100 × 100 × 400 mm prisms from a four-point flexural tensile test.

HRB400 steel bars were used for tension reinforcement; the mean yielding strength, ultimate strength, and modulus of elasticity of the bars are shown in Table 5.

### 2.3. Manufacturing of Beam

**Figure 2:**
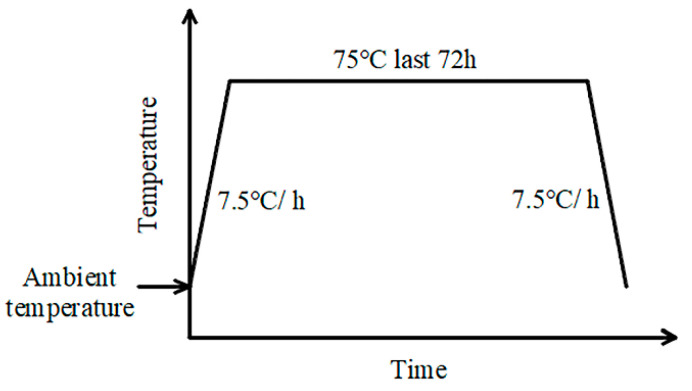
Temperature controlling process of steam-curing for UHPC layer.

The production processes for UHPC-NC composite beams are as follows:

(1) The reinforcing bars would be embedded in the UHPC layer, and all the vertical stirrups of the beam were constructed. The steel formwork was then assembled.
(2) UHPC was poured into the formwork layer by layer until the design thickness of the UHPC layer was reached. The top surface of the UHPC layer was scratched without scraping after vibration in order to maintain the roughness of the top surface of the UHPC layer.
(3) Beams were placed into the curing pool after 6 h of standing. A 72 h steam cure at a temperature of 75 °C was adopted for the beam, as shown in Figure 2.
(4) The reinforcing bars within the NC layer were constructed after the steam-curing of the UHPC layer, and the top surface of the UHPC layer was moistened before casting the NC. Considering all the test beams were prepared and cured in winter at a low ambient temperature, beams were removed from the steel formwork after 500 min of steam-curing at a temperature of 50 °C.

Beams were kept in the plant before the experiments started for longer than one year, thus the impact of autogenous shrinkage and creep of NC or UHPC on the experimental results throughout the testing period could be diminished.

**Figure 3:**
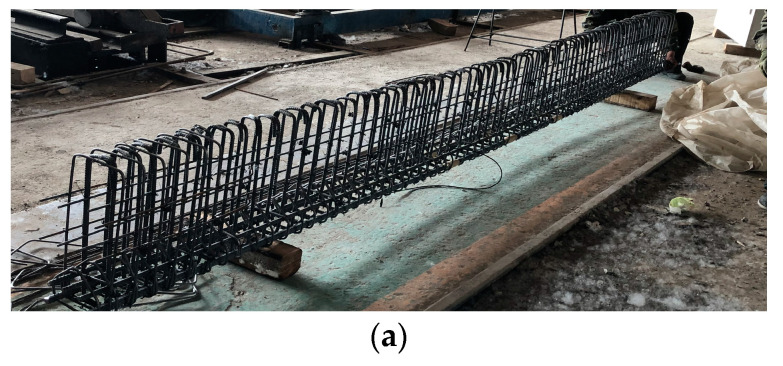

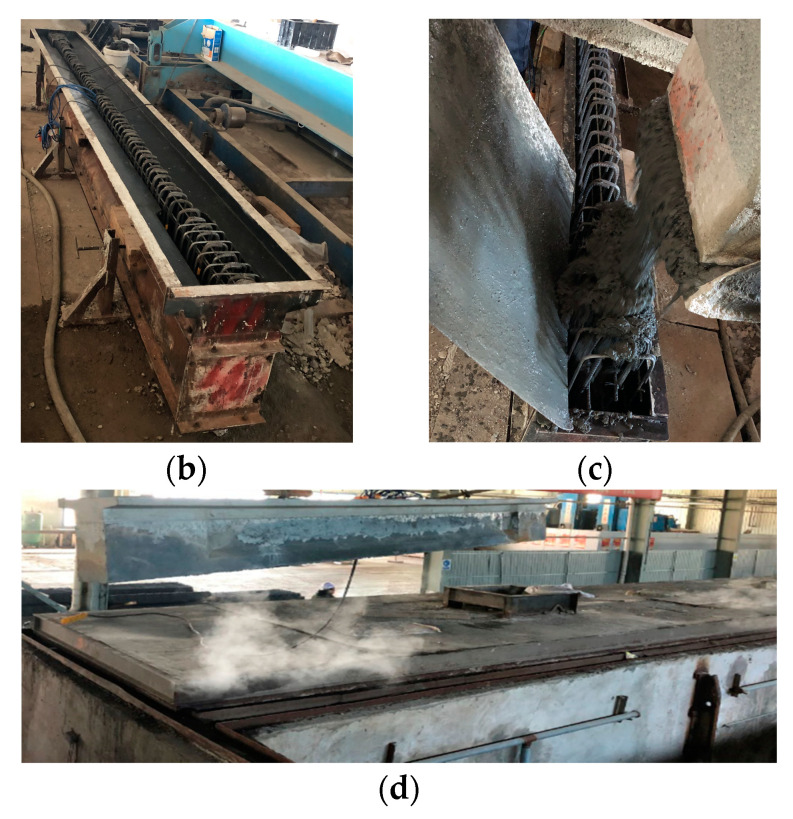
Fabrication process of UHPC-NC composite beams. (**a**) Constructing the reinforcement skeleton; (**b**) Assembling the steel formwork; (**c**) Pouring the UHPC; (**d**) Steam-curing in the pool.

In this process, UHPC and NC specimens tested for evaluating the mechanical properties of concrete materials were poured and cured together with their corresponding test beams. The fabrication process for test beams is shown in Figure 3.

### 2.4. Experimental Program

**Figure 4:**
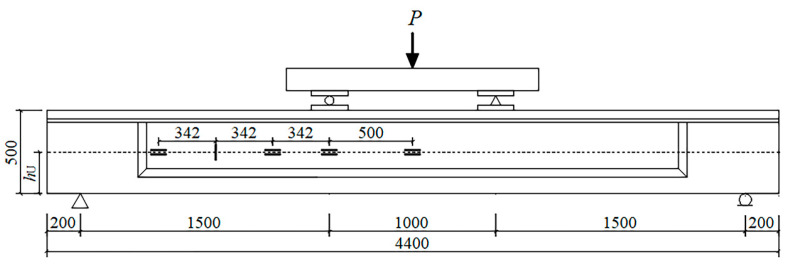
Loading layout of static and fatigue tests (mm).

All beams were simply supported with an effective span of 4000 mm and tested in four-point bending using an Instron servo-controlled hydraulic system. A typical test setup is shown in Figure 4.

Fatigue tests were carried out in load control with a sinusoidal wave. Fatigue loading frequencies of 2–3 Hz were adopted in this study and barely affected the fatigue behavior of the beam in this frequency range. For saving time and cost, the loading cycles were repeated until the beam failed, or stopped after 2,000,000 cycles and declared a run-out. In order to acquire the effect of loading cycles on the bending performance of the beam, the actuator was programmed to periodically pause throughout fatigue tests, and subsequently, the beam was monotonically loaded to the upper loading. The static tests were initially conducted at short intervals (about tens of thousands of cycles), while the intervals were gradually increased to hundreds of thousands of cycles.

The appearance of the delamination crack at the interface between UHPC and NC layers was recorded in several studies [46,47], and deformation compatibility between the two layers in a composite beam would be weakened by the interfacial debonding. In order to verify the deformation compatibility between UHPC and NC layers in the shear span, three pairs of strain gauges were attached parallelly on the two sides of the interface between NC and UHPC layers at a distance of 0, 500, 842, and 1526 mm from the middle span, which were labeled as “material of the layer—horizontal distance from the position of the strain gauge to the midspan”. In addition, a strain gauge labeled “Vert-1184” was attached perpendicularly across the interface at a distance of 1184 mm from the middle span (except for beam L-0-55) for monitoring the vertical strain subjected to the interface in the shear span. The locations of strain gauges can be seen in Figure 4. It should be noted that there was around a 20 mm height difference between the positions of each pair of strain gauges (the two strain gauges in UHPC and NC are at the same distance from midspan), limited by the size of the strain gauges.

## 3. Results and Discussion

### 3.1. Fatigue Life of the UHPC-NC Composite Beam

The fatigue lives of beams are listed in Table 6. It can be seen that the fatigue life (*N*_f_) of the UHPC-NC composite beam was prolonged as the thickness of the UHPC layer increased. Although beams L-0-55, L-200-55, and L-360-55 had the same reinforcement ratio and were subjected to identical upper and lower load limits, which ranged from 25.0 kN to 172.0 kN, beams could sustain substantially different cycles in the fatigue tests owing to the difference in UHPC layer thicknesses. The fatigue life of beam L-200-55 with a 200 mm thick UHPC layer was approximately 5.2 times that of beam L-0-55, and the fatigue life increased by 53% as the UHPC layer thickness increased from 200 mm to 360 mm. Based on the results, it can be assumed that there is an optimum value of the *h*_U_/*h* ratio (UHPC layer thickness/height of the composite beam) for the UHPC-NC composite beam to maximize the benefits of the UHPC layer on the fatigue resistance of the composite beams. Further studies on a wider scope of *h*_U_/*h* ratio should be conducted to analyze the relationship between *h*_U_/*h* ratio and the fatigue life improvement of the UHPC-NC composite beam. Despite the fact that the relationship between fatigue life and UHPC layer thickness is not clear according to the fatigue test results in Table 6, it can be clearly seen that the UHPC layer has a beneficial effect on prolonging the fatigue life of the beam due to its good tensile resistance and energy absorption capacity.

### 3.2. Interface Performance under Static Loading

Bending tests were conducted on twelve 500 mm-height UHPC-NC composite beams [48], with relatively thinner (150, 200, and 260 mm) UHPC layers, and experimental results showed that the delamination crack propagated through the interface between UHPC and NC layers in the shear span for the beams with a 150 mm-thick UHPC layer. However, another ten UHPC-NC composite beams with a 500 mm height and relatively thicker (290, 360, and 430 mm) UHPC layer [49] were monotonically loaded to failure as well; there was no interfacial crack between the UHPC and NC layers observed until the ultimate bearing capacity of the beam was reached. It is worth emphasizing that the dimensions of beams in the research [49] were the same as those of beams in our study.

**Figure 5:**
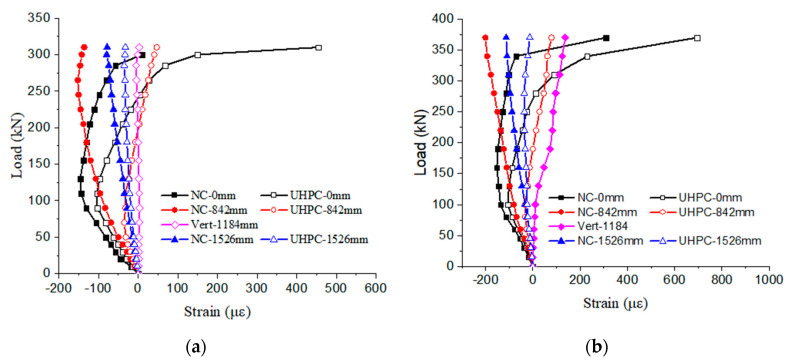
Strain variations at the interface between UHPC and NC layers of beams under monotonic loading: (**a**) L-360-S; (**b**) H-360-S.

For the beams under monotonic loading in our study, the strain variations at the interface between UHPC and NC layers are shown in Figure 5, plotted against the loads. As can be seen from the figures, strains in the shear-span of the beam slowly develop with increasing load, and those of “NC/UHPC-842 mm” and “NC/UHPC-1526 mm” are all at a low level. More specifically, the tensile and compressive strains are less than 79 με and 201 με, respectively, for beam H-360-S, and the differences between each pair of strains increase slowly, and the maximum difference reaches 280 με as the bending failure approaches. For beam L-360-S, the tensile and compressive strains are less than 47 με and 136 με, respectively; the maximum difference between these pairs of strains is up to 183 με. Moreover, the maximum stain of “Vert-1184 mm” does not exceed 136 με for the two beams during static tests.

As shown in Figure 5, the strain of the NC layer bottom at midspan is labeled “NC-0 mm” (solid squares), and that of the UHPC layer top at midspan is “UHPC-0 mm” (open squares). Both of them were in the pure bending zone, initially with compressive strain and gradually increasing until the loads arrived at around 90 kN and 100 kN for beams L-360-S and H-360-S, respectively. However, these are also the inflection points of curves; as the loads continue to increase, compressive strains decrease and change to tensile strains. Due to the fact that the position of “NC-0 mm” is higher than that of “UHPC-0 mm”, the former bears a relatively higher compressive strain in the early loading process and a relatively lower tensile strain after the inflection point. According to the curves in the figures, the differences between the pairs of strains at the interface do not go beyond 444 με and 385 με for beams L-360-S and H-360-S, respectively.

In general, the trends of each of these pairs of strains at the interface were basically similar until the ultimate bearing capacity of the composite beam was reached. The measurement results of strains were consistent with the actual observations, in which no interfacial crack was observed before the beam failed. Considering the 20 mm height difference between the two positions, the actual differences between each pair of strains at the interface would be smaller than the measured values. As a result, it is believed the bond performance at the interface was barely deteriorated for the UHPC-NC composite beams under static loading prior to bending failure.

### 3.3. Interface Performance under Cyclic Loading

In the case of UHPC-NC composite beams being monotonically loaded in bending, the experimental conclusions reported in previous studies have shown that the bond performance between UHPC and NC layers is likely to be affected by UHPC layer thickness [40,41]. Further analysis of the interface performance of the UHPC-NC composite beams under cyclic loading was discussed in detail below.

**Figure 6:**
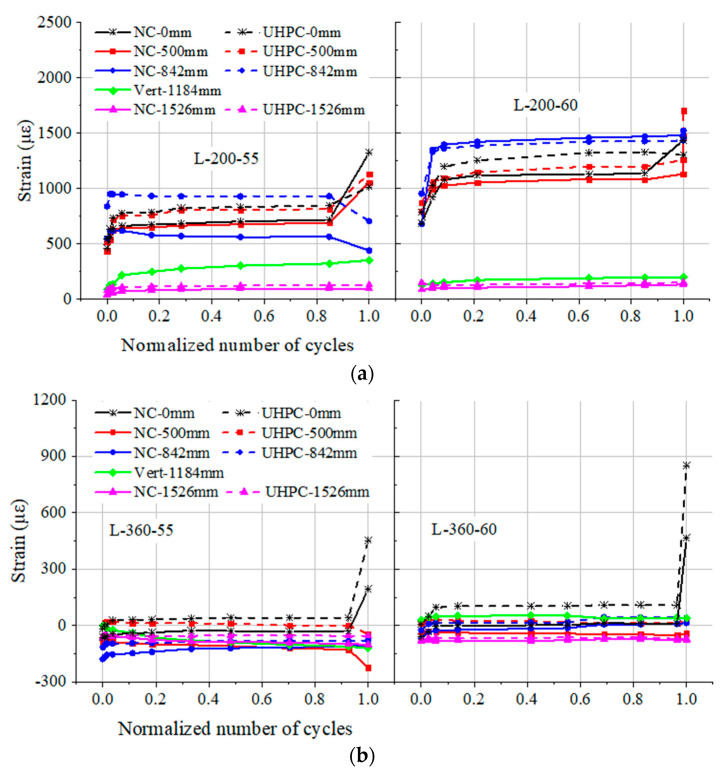
Strain at the interface between UHPC and NC layers versus normalized number of cycles for beams with different thicknesses of UHPC layer: (**a**) L-200-55 and L-200-60; (**b**) L-360-55 and L-360-60.

The strain variations on both sides of the interface for two groups of UHPC-NC composite beams are compared in Figure 6 and plotted against the normalized number of cycles (*N*/*N*_f_). Strain variations in beams with a 200 mm thick UHPC layer are shown in Figure 6a, and those with a 360 mm thick UHPC layer are shown in Figure 6b. The load ranges of 25~172.0 kN and 25~187.6 kN were adopted for beams H/L-200-55 and H/L-360-60, respectively. As can be seen from the figures, the rapid increase or decrease in strain is observed as the normalized number of cycles approaches 1.0. This may be explained by the fact that there is a rapid propagation of cracks as failure occurs. However, prior to the failure of beams, each pair of strains at the interface had a similar variation tendency.

Because the height of the interface in beams L-200-55 and L-200-60 is lower, NC and UHPC at the interface are subjected to tensile strain during the tests. In contrast, beams L-360-55 and L-360-60 have a relatively thick UHPC layer; thus, NC and UHPC at the interface mainly bear compressive strain in initial cycles and thereafter have a very limited decrease in the following cycles.

The strain variation at the interface in beams with a 360 mm thick UHPC layer is significantly less than that with a 200 mm thick UHPC layer; this may be attributed to the fact that the interface of the former lies near the neutral axis of the beam. In addition, the strain at the interface of beam L-200-55 is relatively lower than that of beam L-200-60, owing to the lower load level. The same phenomenon is observed between beams L-360-55 and L-360-60. The strains of “Vert-1184 mm” are at a low level and do not exceed 54 με and 355 με during the whole fatigue life for beams with 360 and 200 mm thick UHPC layers, respectively. It is worth noting that the curves of strain variations in the constant bending moment zone consist of three typical stages: there is an obvious increase in the initial stage, followed by a relatively stable stage, and a rapid increase in the third stage (there was a premature failure of the strain gauge at mid-span of beam L-200-60). However, strains at the interface within the shear span of the beam show much less variation and basically remain steady during the fatigue tests.

As mentioned above, there was practically no interfacial crack at the interface and barely any reduction in the interface performance between the NC and UHPC layers for the composite beam prior to fatigue failure. This Means that a good bonding characteristic could be provided at the interface and the UHPC layer could act in conjunction with the NC layer for the composite beam under repeated loading. Based on the results of this experiment, it can be argued that for the UHPC-NC composite beam with a *h*_U_/*h* ratio of not less than 40%, the interface between UHPC and NC layers is not the weakness of the composite beam subjected to cyclic loading.

### 3.4. Degradation of Bending Stiffness

The deflection variation of UHPC-NC composite beams under fatigue loading can be divided into three stages: initially rapid accumulation, →gradually increasing with the increase in the number of cycles, →significantly growing before rupture, which has been described in detail in our previous study [50]. The bending stiffness of the beam can be calculated using Equation (1):

(1) $$B=\frac{f(M,l)}{D}$$

In the above, *B* is the bending stiffness of the beam, *M* is the moment applied in the pure bending zone of the beam, *l* is the span, and *D* is the deflection at the midspan of the beam.

The deflection accumulation of the beam during fatigue tests indicates that the bending stiffness is reduced with an increasing number of cycles. Accordingly, the stiffness reduction coefficient, *λ*, can be defined as the ratio of bending stiffness at *N* cycles to that at the first cycle and can be expressed as follows [51]:

(2) $$\lambda =\frac{B_{N}}{B_{1}}$$

where *B*_1_ is the bending stiffness of the beam at the first cycle, and *B_N_* is the bending stiffness at *N* cycles.

**Figure 7:**
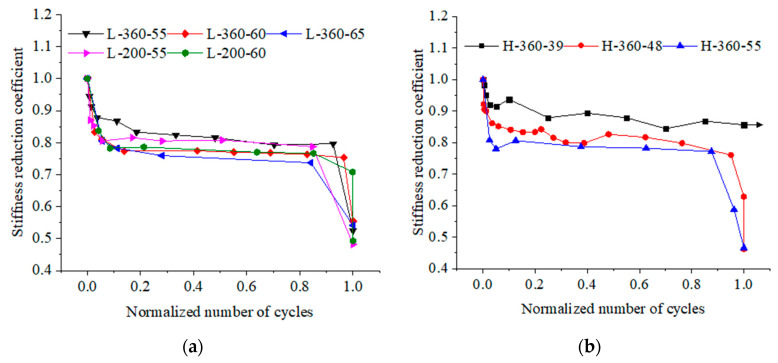
Stiffness reduction coefficient versus normalized number of cycles for beams in (**a**) series L and (**b**) series H.

Figure 7 shows the variations of the stiffness reduction coefficient for beams in two series and is plotted as a function of the normalized number of cycles.

The typically S-shaped curves of stiffness reduction coefficient are similar and can be obviously characterized by three stages: stage I experiences an obvious decrease in less than 10% *N*_f_; unrecoverable deformation rapidly accumulates over cycles; stage II keeps a steady decrease during about 75~80% *N*_f_; stage III shows another rapid decrease and occupies around 10~15% *N*_f_. Except for beam H-360-39 (it is run-out), in terms of the beams cyclically loaded to failure, the bending stiffness is decreased to around 80% of its initial value at the end of the first stage (the first inflection point of curves). Afterward, the bending stiffness is slowly degraded during the second stage, which occupies most of the fatigue life; the bending stiffness is gradually decreased to about 75% of the initial value at the end of the stage (the second inflection point of curves). Finally, an evident decrease in bending stiffness is induced by the rupture of reinforcement.

**Figure 8:**
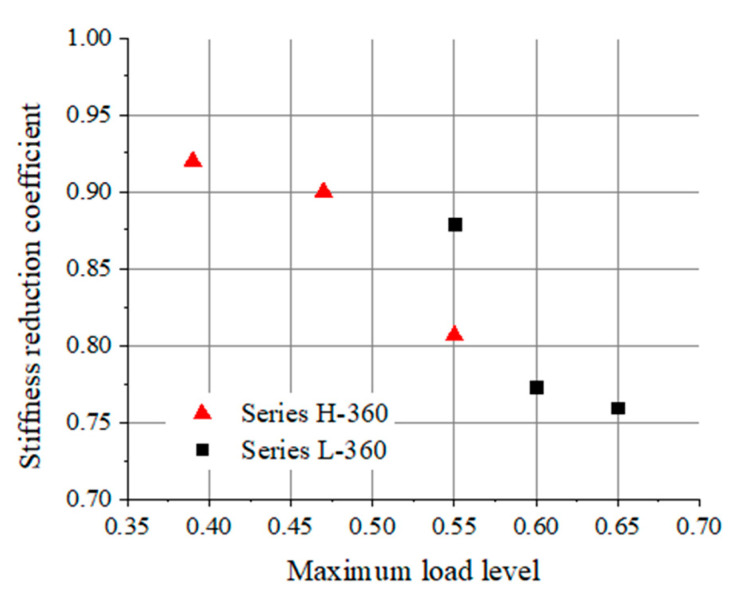
Stiffness reduction coefficient for beams under different load levels after 50,000 cycles.

A higher upper load limit leads to a lower stiffness reduction coefficient for beams, as shown in Figure 8. For the beams of series L with a 360 mm thick UHPC layer, after 50,000 repeated loading cycles, the bending stiffness decreases 12.1% when the upper load limit increases from 0.55 *P*_u_ to 0.60 *P*_u_ (the upper load limit increases 9.1%), and that decreases 13.7% when the upper load limit increases from 0.55 *P*_u_ to 0.65 *P*_u_ (the upper load limit increases 18.2%). This indicates that the stiffness reduction of the beam can be accelerated at higher load levels.

On the other hand, for the beams subjected to an upper load level of 0.55 *P*_u_ and cyclically loaded with 50,000 cycles, the stiffness reduction coefficient for beam L-360-55 is approximately 0.88; however, the corresponding value for beam H-360-55 is 0.81. This decrease can be concluded from the fact that the bending stiffness could decrease more rapidly for the beam with a higher reinforcement ratio under repeated loading. The further reason may be that the contribution of the UHPC layer to the fatigue behavior of the composite beam is enhanced by reinforcing bars; as a result, the greater fatigue resistance of the UHPC-NC composite beam can be attributable to a higher reinforcement ratio.

**Figure 9:**
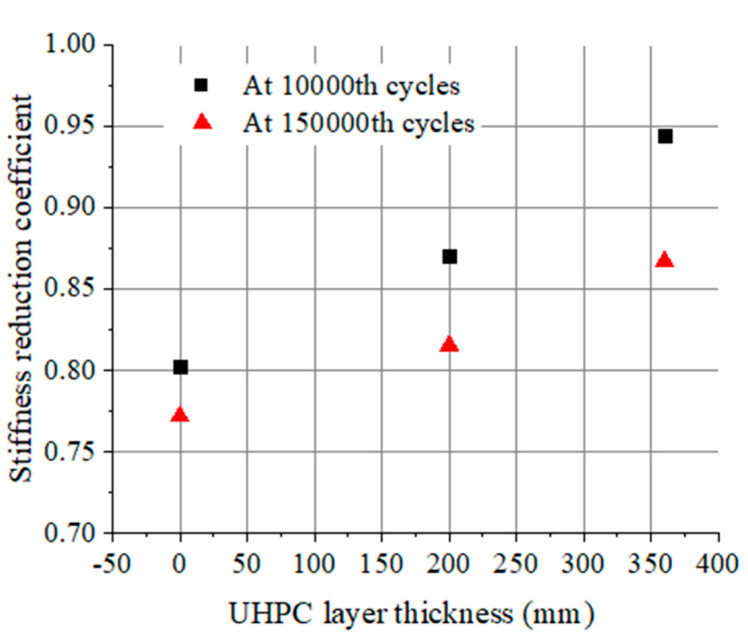
Beams with different UHPC layer thicknesses at 10,000th and 150,000th cycles.

Furthermore, the effect of UHPC layer thickness on the stiffness reduction coefficient is illustrated in Figure 9. There is basically a positive correlation between the stiffness reduction coefficient of the beam and the UHPC layer thickness. The stiffness reduction coefficients for beams L-200-55 and L-360-55 are compared, showing that after 150,000 repeated loading cycles, their stiffnesses increase by 6.5% and 13.0% relative to the corresponding value for beam L-0-55, respectively. This indicates that the bending stiffness of the UHPC-NC composite beam is improved by increasing the UHPC layer thickness, which rises nonlinearly with the increase in UHPC layer thickness.

According to the discussion above, the conclusions can be generalized as follows: the stiffness reduction process of the beam is affected by the reinforcement ratio, UHPC layer thickness, and fatigue load levels under cyclic loadings.

**Figure 10:**
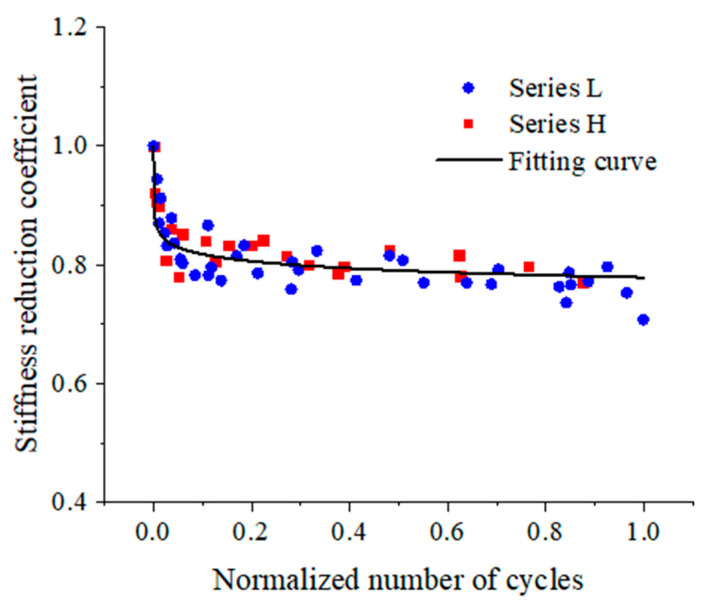
Fitting curve of stiffness reduction coefficient for beams.

The stiffness reduction coefficients for beams in series L and H are all plotted in Figure 10. A cycle-dependent equation of the stiffness reduction coefficient is obtained by fitting the experimental results and can be written as follows:

(3) $$\lambda =0.7789-0.017\ln\left(\frac{N}{N_{f}}\right)$$

The correlation coefficient of Equation (3) is *R*^2^ = 0.8533.

Subsequently, a corresponding fitting curve is plotted in Figure 10 as well. It can be seen from the figure that the fitting results are in good agreement with the experimental results.

## 4. Conclusions

This paper is focused on the effect of the UHPC layer, reinforcement configuration, and load level on the fatigue performance of UHPC-NC composite beams. A series of monotonic and cyclic tests were carried out. The following conclusions could be drawn:

1. Compared to the RC beam, the fatigue lives of UHPC-NC composite beams were increased by 4.2 and 7.0 times for the beams with a 200 and 360 mm-thick UHPC layer, respectively.
2. The variation of each pair of strains at the interface between UHPC and NC layers kept a similar trend until the beam failed to bend; this indicated the interface performance of the UHPC-NC composite beam barely deteriorated under static loading.
3. Under repeated loading, UHPC-NC composite beams showed practically no interfacial cracking and a reduction in interface performance prior to fatigue failure. With a *h*_U_/*h* ratio greater than 40%, the interface between NC and UHPC layers would not be a weakness for the composite beam subjected to cyclic loading.
4. The curves of stiffness reduction coefficient for the composite beams could be characterized by three stages: stage I experienced a rapid decrease in less than 10% *N*_f_; stage II kept a steady decrease during about 75~80% *N*_f_; stage III showed another rapid decrease and occupied around 10~15% *N*_f_. Moreover, a calculation method for the stiffness reduction coefficient of UHPC-NC composite beams under cyclic loading was proposed based on the experimental results.

## Figures and Tables

**Table 1:** Parameters of beams.

Test Series	Beam Designation	UHPC Layer Thickness (*h*_U_)/mm	Reinforcement Ratio (*ρ*_s_)/%
Series S	L-360-S	360	1.184
	H-360-S	360	1.786
Series L	L-0-55	0	1.184
	L-200-55	200	1.184
	L-200-60	200	1.184
	L-360-55	360	1.184
	L-360-60	360	1.184
	L-360-65	360	1.184
Series H	H-360-39	360	1.786
	H-360-48	360	1.786
	H-360-55	360	1.786

**Table 2:** Composition of the UHPC and NC.

UHPC	Cement	Silica fume	Quartz sand	Crushed stone	Water	Superplasticizer	Steel fiber
	1.00	0.285	1.258	0.877	0.256	0.006	0.192
NC	Cement	Mineral powder	River sands	Crushed stone	Water	Superplasticizer	
	1.00	0.098	1.719	2.613	0.336	0.013	

**Table 3:** Properties of steel fiber.

Type	Average Length (mm)	Diameter (mm)	Aspect Ratio	Ultimate Tensile Strength (MPa)
Copper-coated steel fiber	13	0.2	65	2860

**Table 4:** Mechanical properties of NC and UHPC.

Material	NC			UHPC		
Beam	Compressive Strength (MPa)	Flexural Tensile Strength (MPa)	Elastic Modulus (GPa)	Compressive Strength (MPa)	Flexural Tensile Strength (MPa)	Elastic Modulus (GPa)
L-360-S	62.24	6.52	37.23	138.80	15.11	45.57
H-360-S	58.96	6.86	37.92	128.41	14.36	45.26
L-0-55	59.12	6.61	37.00	-	-	-
L-200-55	60.10	6.65	35.57	134.07	14.51	46.46
L-200-60	60.10	6.65	35.57	134.07	14.51	46.46
L-360-55	64.85	6.70	38.26	130.48	14.21	44.07
L-360-60	64.85	6.70	38.26	130.48	14.21	44.07
L-360-65	62.24	6.52	37.23	138.80	15.11	45.57
H-360-39	57.49	6.58	37.53	129.63	14.67	43.38
H-360-48	57.49	6.58	37.53	129.63	14.67	43.38
H-360-55	58.96	6.86	37.92	128.41	14.36	45.26

**Table 5:** Mechanical properties of reinforcements.

Diameter (mm)	Yield Strength (MPa)	Ultimate Strength (MPa)	Modulus of Elasticity (GPa)
12	449	607	201
14	455	627	200
16	456	616	200
20	441	615	201

**Table 6:** Fatigue test results.

Beam Designation	*P*_max_ ^1^	*P*_min_ ^2^	Frequency (Hz)	*N*_f_
L-0-55	0.55	0.08	4.0	169,235
L-200-55	0.55	0.08	4.0	884,707
L-200-60	0.60	0.08	3.0	234,917
L-360-55	0.55	0.08	4.0	1,350,708
L-360-60	0.60	0.08	3.0	362,802
L-360-65	0.65	0.08	2.0	178,241
H-360-39	0.39	0.16	4.0	2,000,000 ^3^
H-360-48	0.48	0.07	3.0	4,258,667
H-360-55	0.55	0.08	2.0	399,698

^1^ *P*_max_: Upper load limit/ultimate bearing capacity. ^2^ *P*_min_: Lower load limit/ultimate bearing capacity. ^3^  Beam H-360-39 did not fail after 2,000,000 load cycles.

## Data Availability

Data are contained within the article.
